# The complete chloroplast genome sequence of *Lilium concolor* var. *pulchellum*

**DOI:** 10.1080/23802359.2022.2048210

**Published:** 2022-09-15

**Authors:** Shengnan Kang, Xuemin Zhang, Mingfang Zhang, Yunpeng Du, Haiying Qin, Xiaonan Yu, Zhang Xiuhai

**Affiliations:** a Beijing Key Laboratory of Ornamental Plants Germplasm Innovation & Molecular Breeding, Beijing Laboratory of Urban and Rural Ecological Environment, College of Landscape Architecture, Beijing Forestry University, Beijing, China; b Institute of Grassland, Flowers and Ecology, Beijing Academy of Agriculture and Forestry Sciences, Beijing, China; c Chongqing Municipal Agricultural School, Chongqing, China

**Keywords:** *Lilium concolor* var*. pulchellum*, *Lilium*, chloroplast genome, phylogenetic analysis

## Abstract

*Lilium concolor* var*. pulchellum* is a perennial herbaceous plant with high ornamental and edible value; it is a critical breeding parent of Asiatic hybrids. In this study, we reported the complete chloroplast genome of *L. concolor* var. *pulchellum*. The total size of the genome is 152,126 bp with a GC content of 37.0%. It has a conserved quadripartite structure comprising 136 genes, including 38 tRNA genes, 8 rRNA genes, 83 protein-coding genes, and 7 pseudogenes. Phylogenetic analysis strongly supported a close relation between *L. concolor* var. *pulchellum* and *L. callosum*. The complete plastome sequence of *L. concolor* var. *pulchellum* could provide useful information for phyletic evolution of the genus *Lilium*.

*Lilium concolor* var. *pulchellum* (Fisch.) Regel 1876, belonging to the genus *Lilium* in the Liliaceae family, is an excellent germplasm resource of the genus *Lilium.* It has a great potential for development and utilization (Du et al. 2014). *L. concolor* var. *pulchellum* is naturally distributed in Northern China (Liaoning, Jilin, Heilongjiang, Hebei province), South Korea, Russia, and North Korea (Liang and Tamuram 2000). At present, the main phylogenetic clades of the genus *Lilium* have been basically defined, and the renewal system could be divided into seven sections (Martagon, Pseudolirium, Lilium, Archelirion, Sinomartagon, Leucolirion, and Oxypetalum) according to the morphological taxonomy and molecular phylogenetic methods (Gao et al. 2013; Du et al. 2017). Some of these groups are subdivided into several subgroups. Sinomartagon is the fifth group, divided into Sinomartagon5a and Sinomartagon5b due to morphological characteristics(Comber 1949; De 1974). In this study, we report the complete chloroplast (cp) genome of *L. concolor* var. *pulchellum* for the first time. It could provide the genomic resource for further studies on the genetic evolution of the genus *Lilium*.

Healthy and fresh leaves of *L. concolor* var. *pulchellum* species were collected from National Lily Germplasm Bank of Beijing Academy of Agriculture and Forestry Sciences, Beijing, China (39°56′37″N, 116°17′15″E). The collection of plant material was carried out in accordance with the guidelines provided by National Lily Germplasm Bank of Beijing Academy of Agriculture and Forestry Sciences and with the permission from the institution. The specimen was stored in the Herbarium, Institute of Botany, Chinese Academy of Sciences (contact Wang Zhongtao, pe@ibcas.ac.cn). The specimen accession number is Z0166. Total genomic DNA was extracted using a plant genome extraction kit (Tiangen, Beijing, China). Subsequently, DNA concentration was measured using a NanoDrop spectrophotometer 2000 (Thermo Fisher Scientific, America). DNA was sheared to construct a 400 bp (insert size) paired-end library by the Illumina HiSeq 4000 standard protocol. The paired-end reads were qualitatively assessed and completed using SPAdes 3.6.1 (Bankevich et al. 2012). The gaps were filled by PCR amplification and Sanger sequencing. The Sanger sequence reads were proofread and assembled with Sequencher 4.10 (http://www.genecodes.com).

All genes encoding proteins, transfer RNAs (tRNAs) and ribosomal RNAs (RNAs) were annotated on *Lilium*. Plastomes were annotated using Dual Organellar Genome Annotator (DOGMA) software and the tRNAscan-SE 1.21 program (Wyman et al. 2004, Schattner et al. 2005). Initial annotation and the putative starts, stops and intron positions were identified by comparison with other *Lilium* cp homologous genes. The complete cp genome of *L. concolor* var. *pulchellum* was submitted to GenBank with the accession number MZ751064.

The length of *L. concolor* var. *pulchellum* chloroplast genome is 152,126 bp. The genome is assembled in the single circular, double-stranded DNA sequences, displaying a typical quadripartite structure, consisting of a pair of Inverted Repeats (IRs, 26,492 bp) separated by the Large Single Copy (LSC, 82,058 bp) and Small Single Copy(SSC, 17 084 bp) regions. The total GC content is 37.0%, and the corresponding values of the LSC, SSC, and IR regions are 34.8, 30.8, and 42.5%, respectively. The chloroplast genome contained 136 genes in total, including 83 protein-coding genes, 38 tRNA genes, 8 rRNA genes, and 7 pseudogenes (*ycf68* (×2), *ycf15* (×2), *ycf1* (×2), and *infA*). In addition, there were 23 genes that contained the introns, three of them (*ycf3*, *rps12*, *clpP*) had two introns, and the rest had only one intron (*rnK-UUU*, *rps16*, *trnG-UCC*, *atpF, rpoC1*, *trnL-UAA*, *trnV-UAC*, *petB*, *petD*, *rpl16*, *rpl2*(*×2*), *ndhB*(*×2*), *trnL-GAU*,*trnA-UGC*, *nDHA, trnA-UGC*,*trnA-GAU*). A total of 64 simple sequence repeats (SSR) were identified in the chloroplast genome of *L*. *concolor* var. *pulchellum* (Beier et al. 2017), ranging from the mononucleotide to pentanucleotide repeat motifs.

To explore the phylogenetic relationship among this species and its related taxa, 17 *Lilium* chloroplast plastome sequences were downloaded from the NCBI Genbank database, using *Cardiocrinum giganteum*, *Notholirion bulbuliferum*, *Notholirion campanulatum,* and *Fritillaria persica* as the outgroup. Phylogenetic trees were constructed by the maximum-likelihood (ML) and Bayesian analysis (BI) methods using the entire cp genome. The ML analyses were performed using RAxML-HPC2 on XSEDE (8.2.12) at the CIPRES Science Gateway website (Stamatakis et al. 2008; Miller et al. 2015) (http://www.phylo.org/sub_sections/portal/), as suggested with 1000 bootstrap replicates. The BI was performed with MrBayes 3.2 (Ronquist et al. 2012). A random tree and 100,000 generations started in the Markov chain Monte Carlo (MCMC).

Phylogenetic analysis showed that *L. concolor* var. *pulchellum* belonged to Sinomartagon 5b, and it formed the closest clade with *L. callosum* with a strong bootstrap support of 100% (Figure 1). The chloroplast genome of *L. concolor* var. *pulchellum* provides the valuable genomic resource for the phylogenetic studies of the genus *Lilium*.

**Figure 1.**
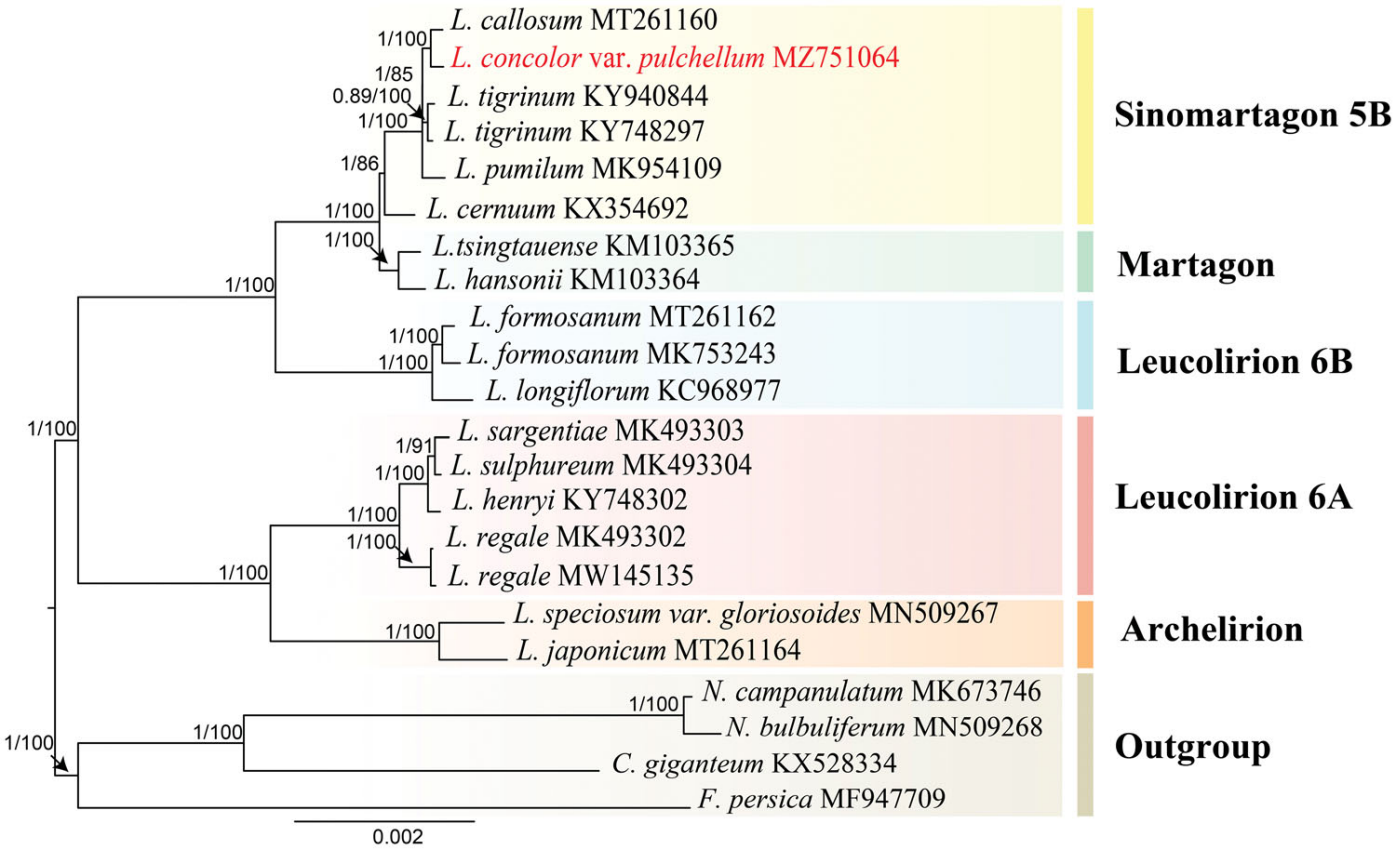
Phylogenetic tree reconstructed by maximum-likelihood (ML) and Bayesian inference (BI) analysis based on chloroplast plastome sequences of *L. concolor* var. pulchellum with 21 species.

## Data Availability

This data has been uploaded to GenBank of NCBI at https://www.ncbi.nlm.nih.gov/genbank, with GenBank accession number MZ751064. The associated BioProject, SRA, and Bio-Sample numbers are PRJNA791705, SRX13501476, and SAMN24338948, respectively.
